# SCDevDB: A Database for Insights Into Single-Cell Gene Expression Profiles During Human Developmental Processes

**DOI:** 10.3389/fgene.2019.00903

**Published:** 2019-09-26

**Authors:** Zishuai Wang, Xikang Feng, Shuai Cheng Li

**Affiliations:** Department of Computer Science, City University of Hong Kong, Kowloon, Hong Kong

**Keywords:** single cell, gene expression, development, database, cell type, differential expression

## Abstract

Single-cell RNA-seq studies profile thousands of cells in developmental processes. Current databases for human single-cell expression atlas only provide search and visualize functions for a selected gene in specific cell types or subpopulations. These databases are limited to technical properties or visualization of single-cell RNA-seq data without considering the biological relations of their collected cell groups. Here, we developed a database to investigate single-cell gene expression profiling during different developmental pathways (SCDevDB). In this database, we collected 10 human single-cell RNA-seq datasets, split these datasets into 176 developmental cell groups, and constructed 24 different developmental pathways. SCDevDB allows users to search the expression profiles of the interested genes across different developmental pathways. It also provides lists of differentially expressed genes during each developmental pathway, T-distributed stochastic neighbor embedding maps showing the relationships between developmental stages based on these differentially expressed genes, Gene Ontology, and Kyoto Encyclopedia of Genes and Genomes analysis results of these differentially expressed genes. This database is freely available at https://scdevdb.deepomics.org

## Introduction

In developmental biology, gene expression changes during the developmental process is an important feature to understand developmental questions such as cell growth, cell differentiation, cell fate decisions, etc. (Ko, 2001; Merks and Glazier, 2005; Gittes, 2009). Recently, high-throughput RNA sequencing technique has been widely used to study gene expression in developmental processes (Spitz and Furlong, 2006). Bulk RNA sequencing typically uses hundreds to millions of cells and reveals only the average expression level for each gene across a large population of cell populations (Wang and Bodovitz, 2010; Sanchez and Golding, 2013). Single-cell RNA-seq measures the distribution of expression levels for each gene across a population of cells and provides a more accurate representation of cell-to-cell variations instead of the stochastic average (Saliba et al., 2014). Therefore, single-cell RNA-seq is particularly apposite for developmental biology (Liu et al., 2014; Griffiths et al., 2018).

High-resolution single-cell transcriptome analysis has been performed during many developmental processes including preimplantation development from oocyte to morula (Xue et al., 2013; Yan et al., 2013), early forebrain and mid/hindbrain cell differentiation from human embryonic stem cells (hESCs) (Yao et al., 2017), and digestive tract development from human embryos between 6 and 25 weeks (Gao et al., 2018), etc. These studies not only revealed many biological features, including developmental processes, signaling pathways, cell cycle, and transcription factor networks but also provided resources to investigate the gene expression patterns during different developmental processes. Therefore, there is a strong need for a web resource that curates and provides single-cell gene expression profiles during different developmental processes.

So far, several web resources for human single-cell transcriptome data have been reported. scRNASeqDB contains 38 datasets covering 200 human cell lines or cell types and 13,440 samples (Cao et al., 2017). The single-cell expression atlas, launched by the European Bioinformatics Institute (https://www.ebi.ac.uk/gxa/sc/home), contains 52 single-cell RNA-Seq studies, consisting of 61,073 cells from 9 different species. The single-cell centric database “SCPortalen” covers 23 human single-cell transcriptomics datasets that are publicly available from the International Nucleotide Sequence Database Collaboration sites (Abugessaisa et al., 2017). PanglaoDB integrated 209 human single-cell datasets consisting of gene expression measurements from cells originating from a common biological source or experiment (Franzén et al., 2019). However, users of these databases can only query gene expression in specific cell types or population heterogeneity processed by the authors. Researchers who are interested in gene expression changes during a specific developmental process are not easily able to extract these dynamic features from these databases.

Here, we developed a database to investigate single-cell gene expression profiling during different developmental processes (SCDevDB). In this database, we collected 10 human single-cell RNA-seq datasets, split these datasets into 176 developmental cell groups, and constructed 24 different developmental pathways. Users of SCDevDB are easy to view the expression changes of their interested genes showed with a boxplot. In addition, users can also download differentially expressed (DE) genes during each developmental pathway, the T-distributed stochastic neighbor embedding (t-SNE) map constructed with these genes, Gene Ontology (GO) and Kyoto Encyclopedia of Genes and Genomes (KEGG) analysis results of these differentially expressed (DE) genes. This database is publicly available at https://scdevdb.deepomics.org. It helps researchers within the fields of developmental biology to facilitate gene expression studies in human single cells.

## Materials and Methods

### Transcriptomic Data Collection

We searched the National Center for Biotechnology Information Gene Expression Omnibus database using the successfully utilized keywords, single-cell RNA-seq, single-cell RNA-seq, single-cell transcriptome, and selected the species to humans. In this study, we only focused on the normal human developmental processes; thus, we abnegated experiments using tumor and other samples treated with chemical reagents. After carefully reviewing the resultant papers and datasets, we obtained 10 datasets for human single-cell RNA-seq using normal cell type, tissue, or organs. These datasets including human cell groups related to the nervous system, digestive system, the heart, the brain, hESC, cell lines, and others. Single cells originating from the same cell lines, tissue regions, or organ regions at the same developmental time points are treated as a cell group. Based on this rule, we classified the 18,413 single cells into 176 cell groups (**Supplemental Table S1**). Cell groups originating from the same cell lines, tissue regions, or organ regions but at different developmental time points were regarded as one developmental stage. Therefore, the 176 cell groups were merged into 35 developmental stages (**Supplemental Table S1**).

### Data Processing and Gene Expression Profiling Analysis

For the selected RNA-Seq experiments, the gene expression matrices were also retrieved from the Gene Expression Omnibus. For cells in datasets where the fragments per kilobase of exon per million reads mapped (FPKM) were available, we computed the TPM for gene i in cell j, according to:

$$TPM_{i}=\left(\frac{FPKM_{i}}{\sum_{j}FPKM_{j}}\right)\;\times 10^{6}$$

This conversion enables the units to be consistent for dataset-to-dataset comparison. Then, for each dataset, we merged cells originating from the same tissue or organ into one file and performed imputation using the R package single-cell analysis via expression recovery with default parameters. Single-cell analysis via expression recovery takes in a matrix and performs library size normalization during denoising step, which can reduce noise including sequencing depth, the number of cells, and cell composition (Huang et al., 2018). We eventually got 176 different files which are consistent with 176 different cell groups.

### Differential Gene Expression and T-SNE Analysis

For each developmental pathway, we merged the expression data of all developmental stages in this pathway into one file. Then, we conducted DE gene analysis between cell groups in the same developmental pathway using Monocle, which will do all needed normalization steps internally, with default parameters (Qiu et al., 2017). We extracted expression data of the DE genes and performed t-SNE analysis with different perplexity for different process (Pedregosa et al., 2011).

### GO and KEGG Enrichment Analysis

The symbol names of DE genes were used as the gene list input into R packages “GOstats” (Falcon and Gentleman, 2006) and “KEGG.db” (Carlson et al., 2016) for GO and KEGG analysis, respectively. We selected the “ontology” parameter as “BP,” “MF,” and “CC” for GO analysis and “pvalueCutoff” parameter as 0.5 for both GO and KEGG analysis. Top 20 significantly enriched GO terms and KEGG terms were selected to show potential functions of DE genes.

### Database Construction

The SCDevDB website was built using the Django Python Web framework (https://www.djangoproject.com) coupled with the MySQL database. The front-end interface was developed based on the Bootstrap open source toolkit (https://getbootstrap.com). The web interactive visualization graphs were developed using Plotly JavaScript Open Source Graphing Library (https://plot.ly/javascript/). SCDevDB was published using the Apache http server and is accessible at https://scdevdb.deepomics.org/.

## Results

### Datasets Summary and the Developmental Tree Construct

**Figure 1:**
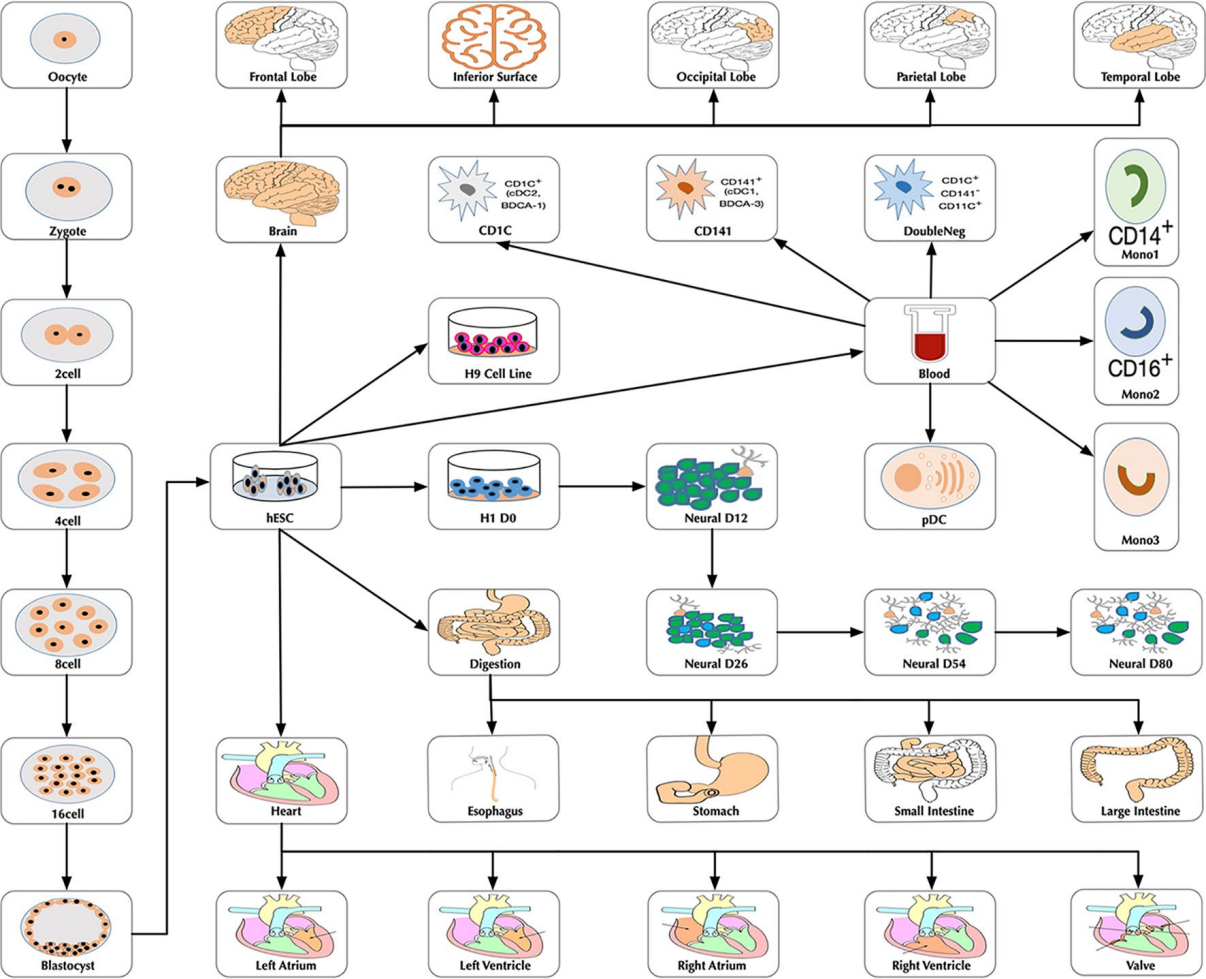
The developmental tree. Figures of the brain, heart and digestion originate from Wikimedia Commons (https://commons.wikimedia.org/wiki/Main_Page).

**Figure 2:**
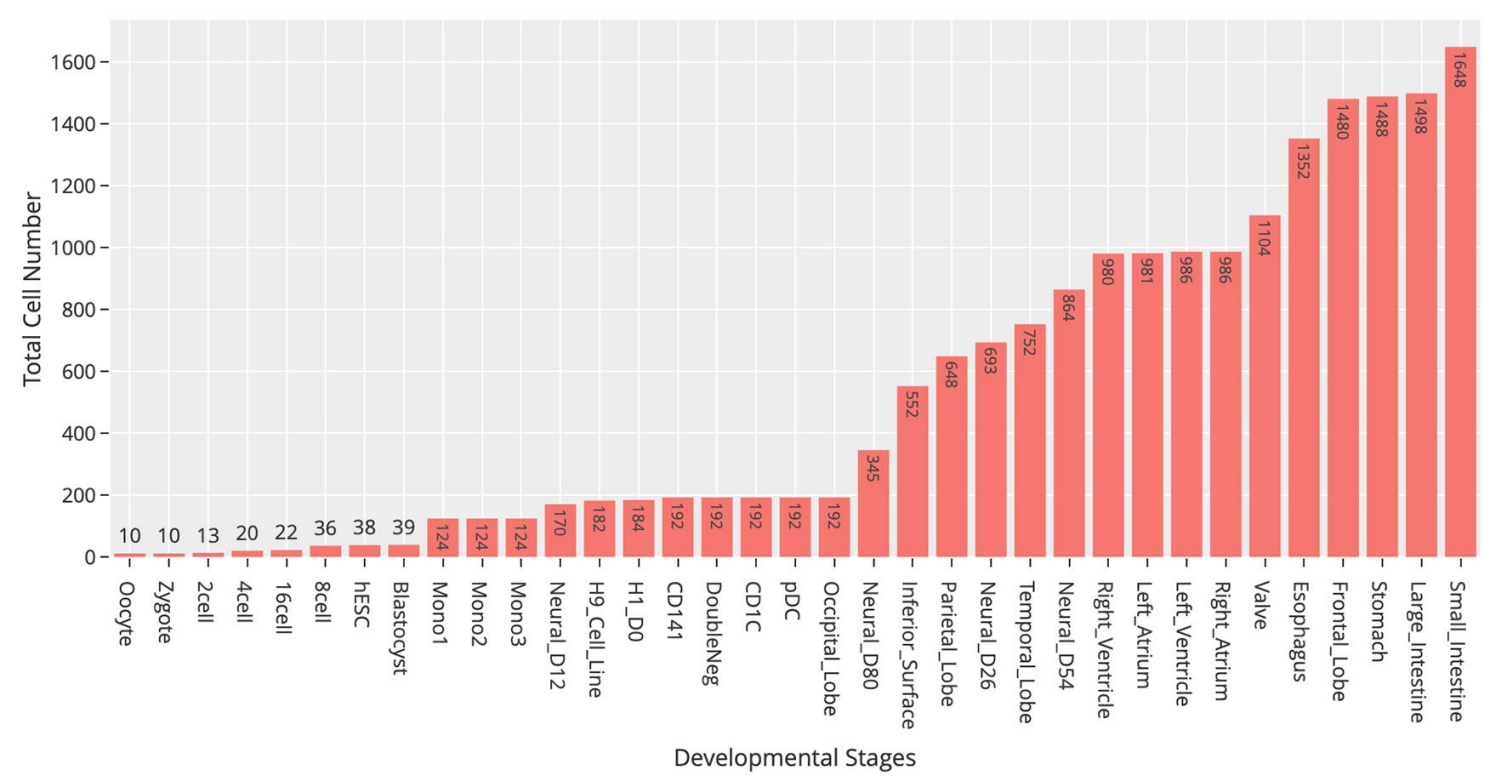
Statistics of cell numbers of 35 developmental stages.

At the time of this publication, the database contains 10 datasets covering 18,413 single cells and 176 cell groups (see Methods). According to the notation of the data resources, we classified these cell groups into 35 developmental stages. Every mammalian individual is developed from the totipotent zygote. Mammalian preimplantation development is a complex process including a series of cell divisions from 1 to 2 cells, 2 to 4 cells, 4 to 8 cells, 8 to 16 cells, and 16 cells to blastocyst (Niakan et al., 2012). After that, nearly all of the human tissues are original from embryoblast (hESC). Then, a developmental tree was constructed based on the development process of the multicellular organism (Hall, 2012) (**Figure 1**). Specifically, we first considered the developmental process from oocyte to hESC as the root process; then, the left 27 developmental stages were classified into 24 different developmental pathways by combining with the root process (**Supplemental Table S1**). The detailed cell number in each stage is shown in **Figure 2**, and the datasets summary is available at https://scdevdb.deepomics.org/data-summary/.

### User Interface to the SCDevDB

**Figure 3:**
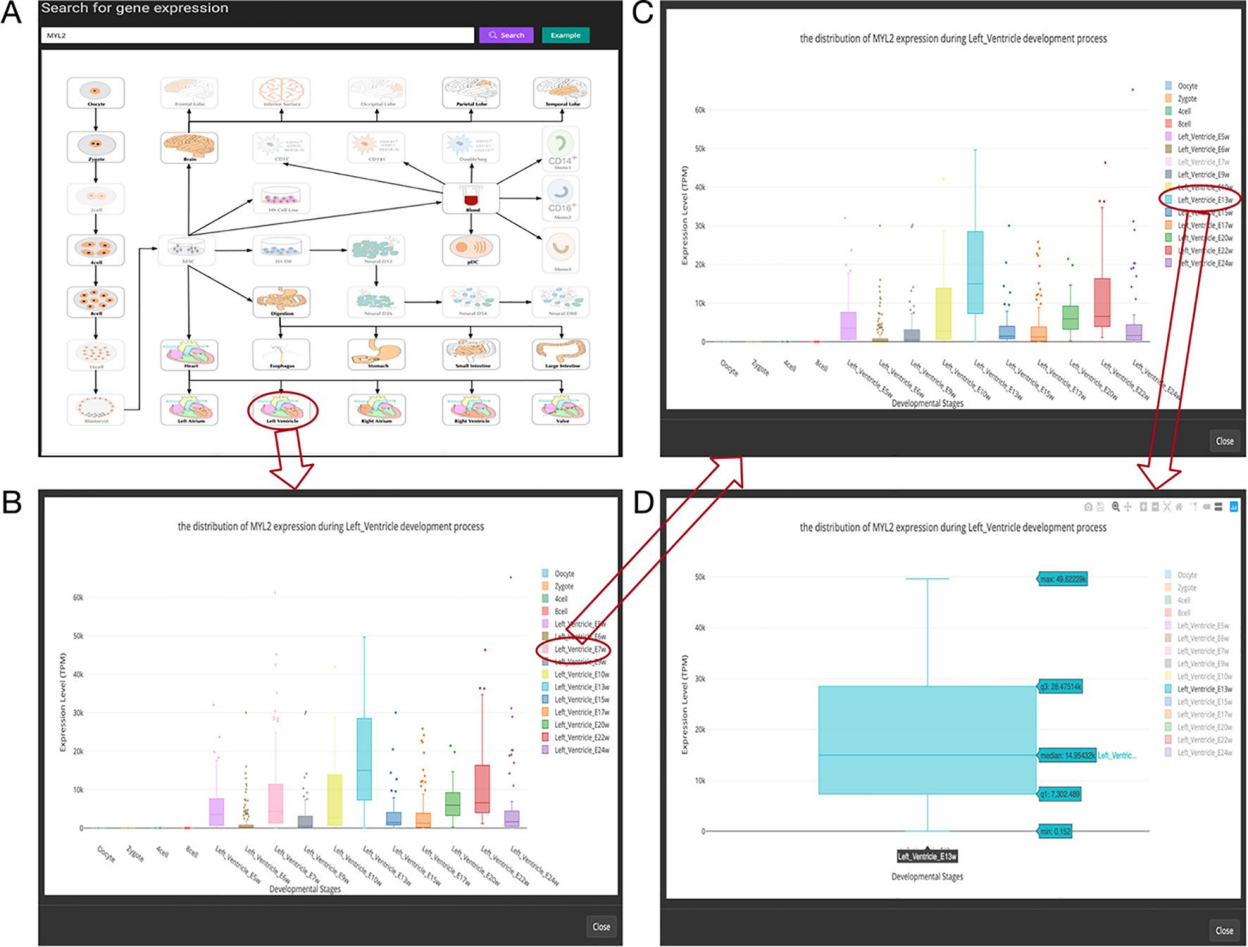
Overview of the gene expression search page. **(A)** Searching result of the gene “MYL2”. **(B)** Boxplot shows expression level distribution of MYL2 during developmental process by clicking the image. **(C)** The function of removing uninterested developmental stages by clicking the name of the stage listed in the figure legend. **(D)** An example of double clicking on a stage name.

**Figure 4:**
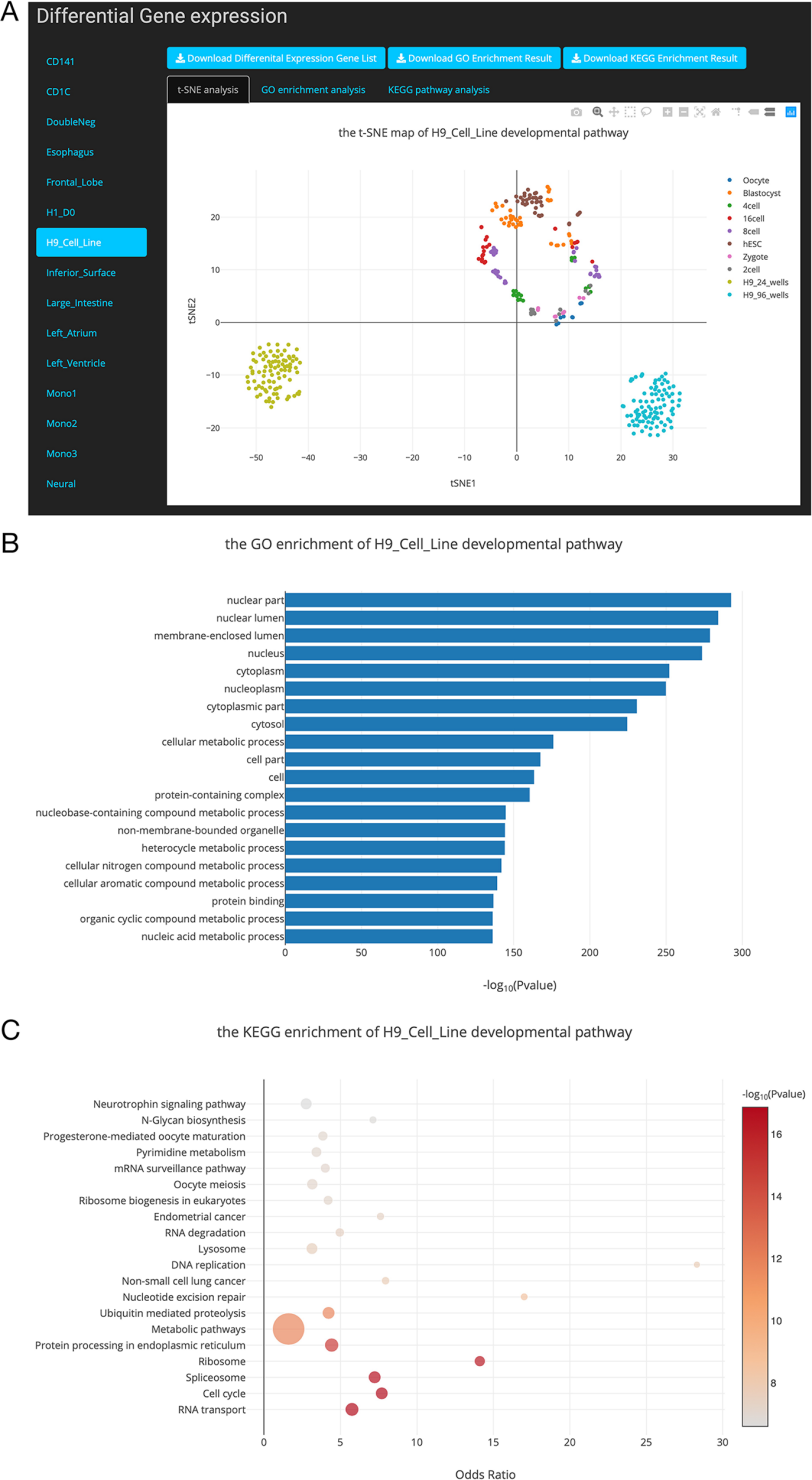
Overview of the differential gene list collection page. **(A)**. T-distributed stochastic neighbor embedding (t-SNE) maps showing the relationships between developmental stages based on these differentially expressed genes. **(B)**. Top 20 Gene Ontology (GO) terms of differential expression genes. **(C)**. Top 20 Kyoto Encyclopedia of Genes and Genomes (KEGG) terms of differential expression genes.

In order to provide users easy access to the SCDevDB, we designed an interface to allow users to perform basic operations, such as searching, viewing, and downloading data. SCDevDB is composed of two functional pages: “Gene Expression Search” page and “Differential Gene List Collection” page. The web interface of SCDevDB is summarized in **Figures 3** and **4**.

### Query Function to Search Gene Expression Across 35 Developmental Stages

In this page, users can view whether an interested gene is expressed in different developmental stages by giving a gene symbol (e.g., APMAP) or an Ensembl ID (e.g., ENSG00000101474) in the searching input box. The searching result will be displayed in the developmental tree. Specifically, if the searched gene (“MYL2” gene as an example) is not expressed at one stage, the stage image will be disabled and cannot be clicked (the light-colored images in **Figure 3A**). Furthermore, the interactive boxplot of gene expression level along with a selected developmental pathway is available by clicking the stage image (**Figure 3B**). To illustrate the interactive function of this boxplot, we took the distribution of the MYL2 expression during left ventricle process as an example. Clicking on the stage name “Left_Ventricle_E7w” listed in the graph legend can remove the boxplot data of this stage (**Figure 3C**). This function allows users to compare their interested stages. Moreover, double clicking on a stage name allows users to view detail gene expression value of this stage (**Figure 3D**). These boxplots can be download in PNG format for further usage.

### Differential Gene List Collection for 24 Developmental Pathways

In this study, we performed DE gene analysis for 24 developmental pathways. Finally, 24 differential gene lists were collected into the SCDevDB. Users can download these gene lists by clicking the Download button in Differential Gene List Collection page. Moreover, we performed t-SNE analysis using these differential gene lists, and the result is displayed using an interactive scatterplot (**Figure 4A**). Subsequently, GO and KEGG enrichment analysis of the DE genes were performed using R packages, and top 20 significantly enriched GO or KEGG terms were selected to show potential functions of these DE genes (**Figures 4B, C**). In addition, tables showing all of the GO or KEGG terms are also available and free to download on the “Differential Gene List Collection” page. These scatterplots and bar chart can be downloaded in PNG format for further usage.

### Case Study

**Figure 5:**
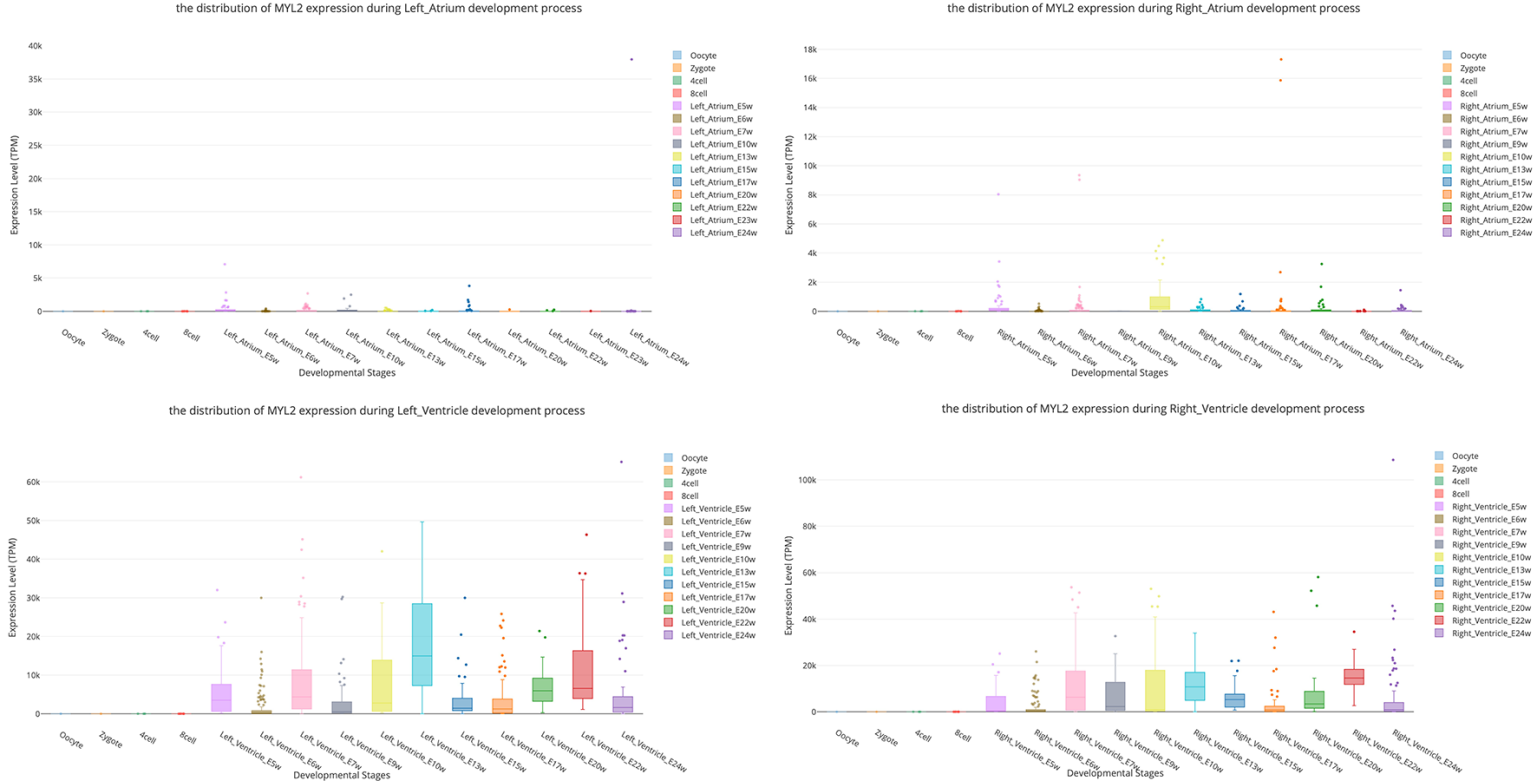
Comparison of MYL2 expression distributions between atrium cell types and ventricle cell types.

Myosin light chain-2 (MYL2, also called MLC-2) is a protein that belongs to the EF-hand calcium binding protein superfamily and exists as three major isoforms encoded by three distinct genes in mammalian striated muscle (Sheikh et al., 2015). Diseases associated with MYL2 include cardiomyopathy, familial hypertrophic, and congenital fiber-type disproportion (Flavigny et al., 1998; Weterman et al., 2013). Here, we used this gene as an interested example to test the functions of SCDevDB. Previous studies using bulk-seq data have shown that MYL2 is highly expressed in tissue of muscles including skeletal muscle, myocardial, and smooth muscles (Hsu et al., 2012; Lindholm et al., 2014; Renaudin et al., 2018). Searching result of the SCDevDB is consistent with these studies as shown in **Figure 3A**. Moreover, comparing with the expression levels in cells of the atriums, MYL2 has higher levels in cells of the ventricles (**Figure 5**). This result indicated that MYL2 can be used as a marker gene to distinguish ventricle and atrium cells in subpopulation analysis.

hESC lines has been used as a source of cells for regenerative medicine, as well as valuable tools for drug discovery and for understanding human development and disease (Allegrucci and Young, 2006). Notably, H9 is one of the first five lines derived in the University of Wisconsin, i.e. H1, H7, H9, H13 and H14 (Denning et al., 2003), which has been used as an important material in many publications (Amit et al., 2000; Gafni et al., 2013; Kim et al., 2014). In our “Differential Gene List Collection” page, when we selected the “H9_Cell_Line” developmental pathway, the t-SNE map indicates that H9 cell lines are distinct from preimplantation cell types (**Figure 4A**). This result is reasonable as the H9 cell line are different from embryonic stem cells in expression levels of various genes (Telugu et al., 2013). Our GO and KEGG analysis results showed that the potential functions of the DE genes during the H9_Cell_Line developmental pathway were enriched in developmental-related biology processes including cellular metabolic process, nucleobase-containing compound metabolic process, RNA transport, and cell cycle pathways.

## Conclusion

In summary, unlike previous databases, SCDevDB is an interactive database providing human single cell resources to profiling gene expression distributions in different developmental pathways. This database also provides DE gene lists in each developmental pathway, t-SNE map, and GO and KEGG enrichment analysis based on these differential genes. We believe that this database will facilitate researchers within the fields of developmental biology to investigate gene expression changes during human developmental pathways in the single-cell level.

## Data Availability Statement

Publicly available datasets were analyzed in this study. These data can be found here: https://www.ncbi.nlm.nih.gov/geo/.

## Author Contributions

ZW performed the data collection and analysis, XF developed the database, SL designed and supervised the study, and ZW, XF, and SL wrote the manuscript.

## Funding

This research was funded by a GRF Project grant from the RGC General Research Fund (9042181; CityU 11203115), the GRF Research Project (9042348; CityU 11257316).

## Conflict of Interest

The authors declare that the research was conducted in the absence of any commercial or financial relationships that could be construed as a potential conflict of interest.
