# Corrigendum: SCDevDB: A Database for Insights Into Single-Cell Gene Expression Profiles During Human Developmental Processes

**DOI:** 10.3389/fgene.2019.01173

**Published:** 2019-11-19

**Authors:** Zishuai Wang, Xikang Feng, Shuai Cheng Li

**Affiliations:** Department of Computer Science, City University of Hong Kong, Kowloon, Hong Kong

**Keywords:** single cell, gene expression, development, database, cell type, differential expression

In the original article, there was a mistake in the legend for **Figure 1** as published. We failed to obtain the relevant permissions from the copyright holders of some figures used in **Figure 1**. The correct figure legend appears below.

“Figure 1. The developmental tree. Figures of the brain, heart and digestion originate from Wikimedia Commons (https://commons.wikimedia.org/wiki/Main_Page).” 

**Figure 1:**
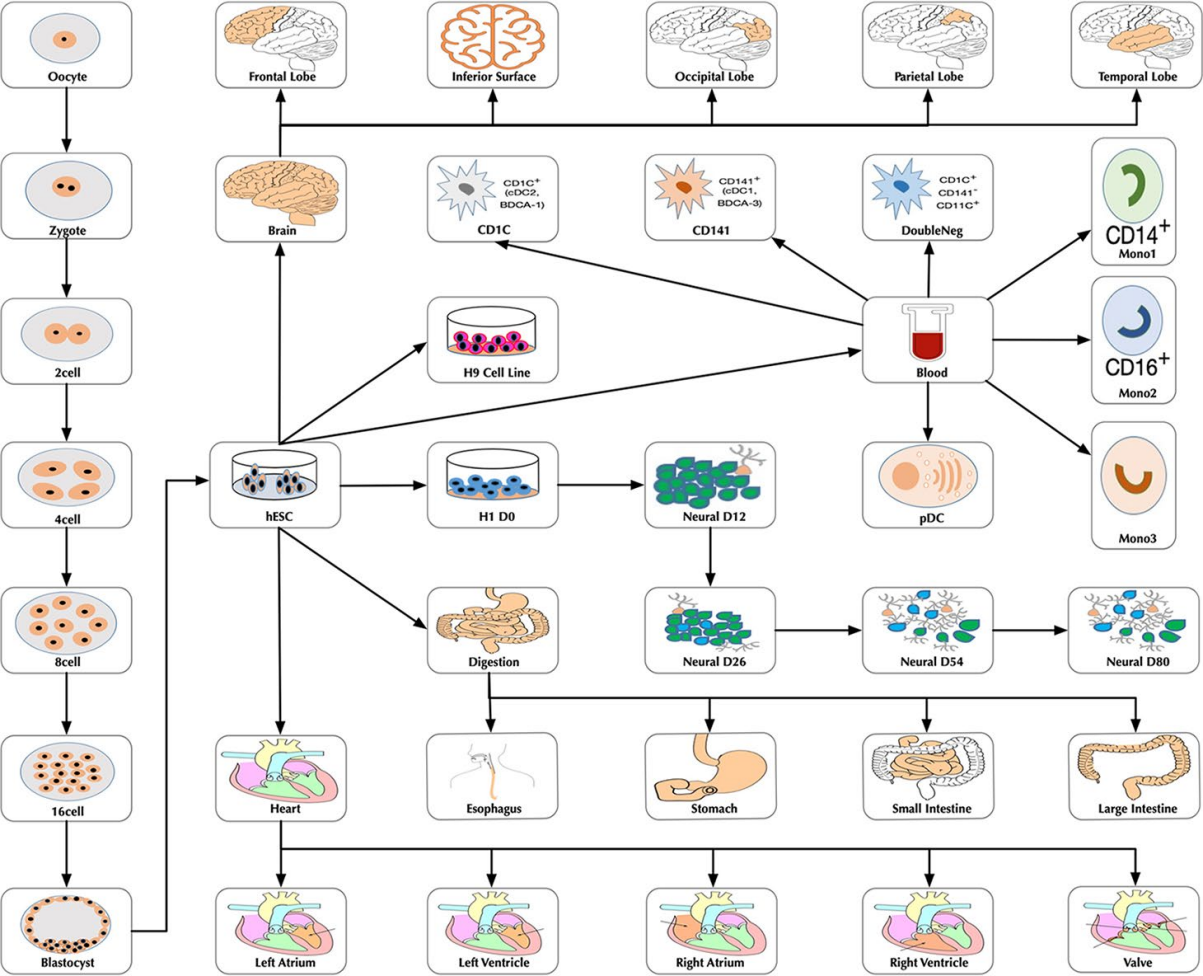
The developmental tree. Figures of the brain, heart and digestion originate from Wikimedia Commons (https://commons.wikimedia.org/wiki/Main_Page).

Additionally, in the original article, there was a mistake in **Figure 1** and **Figure 3** as published. We updated **Figure 1** as we failed to obtain the relevant permissions from the copyright holders of some images used in **Figure 1**, and **Figure 3A** is a thumbnail of **Figure 1**. The correct **Figure 1** and **Figure 3** appear below.

**Figure 3:**
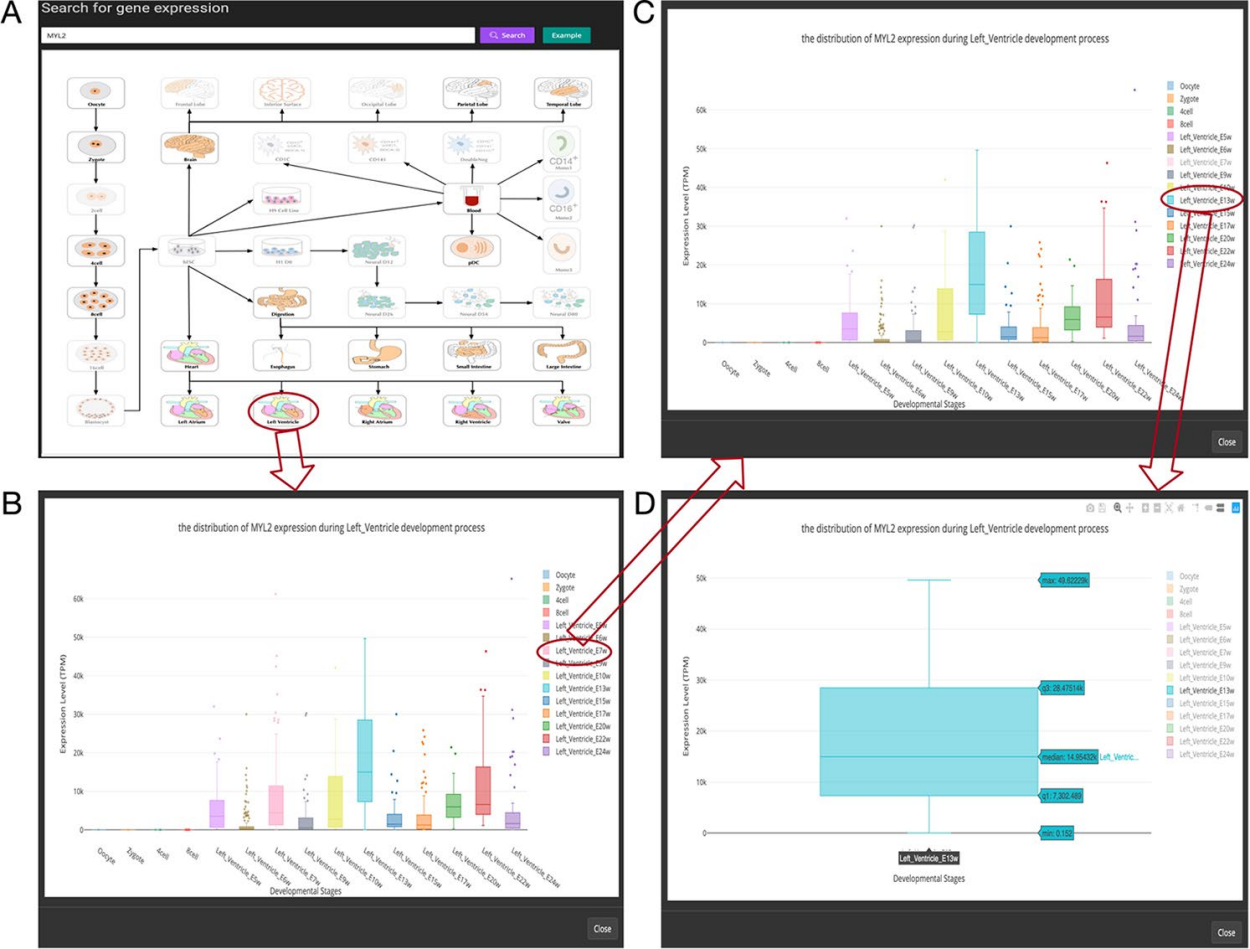
Overview of the gene expression search page. **(A)** Searching result of the gene “MYL2”. **(B)** Boxplot shows expression level distribution of MYL2 during developmental process by clicking the image. **(C)** The function of removing uninterested developmental stages by clicking the name of the stage listed in the figure legend. **(D)** An example of double clicking on a stage name.

The authors apologize for this error and state that this does not change the scientific conclusions of the article in any way. The original article has been updated.

